# A conceptual agentic AI architecture for MASLD-associated significant fibrosis in primary care

**DOI:** 10.1371/journal.pdig.0001500

**Published:** 2026-06-25

**Authors:** Basile Njei, Ulrick Sidney Kanmounye

**Affiliations:** 1 Section of Digestive Diseases, Department of Medicine, Yale University, New Haven, Connecticut, United States of America of America; 2 VA Connecticut Healthcare, West Haven, Connecticut, United States of America of America; 3 Engelhardt School of Global Health and Bioethics, Euclid University, Bangui, Central African Republic; 4 Ohio University Heritage College of Osteopathic Medicine, Athens, Ohio, United States of America of America; 5 Yale Liver Center, Yale New Haven Health, New Haven, Connecticut, United States of America of America; 6 Research Department, Association of Future African Neurosurgeons, Yaounde, Cameroon; Liverpool John Moores University - City Campus: Liverpool John Moores University, UNITED KINGDOM OF GREAT BRITAIN AND NORTHERN IRELAND

## Abstract

Metabolic dysfunction-associated steatotic liver disease (MASLD) is highly prevalent yet often underdiagnosed or undertreated in primary care due to asymptomatic early disease, uneven uptake of non‑invasive tests, limited elastography access, competing clinical priorities, and persistent challenges in sustaining lifestyle modification even after risk is recognized. This opinion introduces ATLAS‑Liver (Adaptive Triage and Learning Agent Suite for Liver disease) as a conceptual reference architecture, not a validated system, for how agentic artificial intelligence (AI) could support guideline‑aligned MASLD pathways by integrating risk estimation, explainability, calibration and fairness monitoring, curated guideline retrieval, and clinician‑retained decision authority within routine workflows. ATLAS‑Liver distinguishes between currently feasible components (e.g., probabilistic models using routine EHR data, local explanation layers with appropriate caveats, subgroup calibration checks, and version‑controlled guideline repositories) and aspirational elements such as dynamic retrieval‑augmented guidance, continuous drift surveillance, and automated agent‑level disagreement resolution. The framework is intended to complement established sequential pathways such as FIB‑4 followed by elastography rather than replace them, offering potential value through improved workflow integration, transparency, follow‑through coordination, and equity monitoring. We situate ATLAS‑Liver within emerging work on AI agents in chronic liver disease while emphasizing its primary‑care orientation and governance‑focused design. We outline key implementation considerations as well as patient‑facing needs such as explanation formats, communication preferences, and support for lifestyle adherence. We acknowledge substantial limitations including lack of empirical validation. ATLAS‑Liver is offered as a hypothesis‑generating framework to guide responsible exploration of agentic AI in primary care MASLD pathways.

## Author summary

Metabolic dysfunction-associated steatotic liver disease (MASLD) affects a large share of adults, yet many patients remain undiagnosed or do not receive timely follow‑up, partly because early disease is silent, clinical visits are time‑pressured, and lifestyle modification is difficult to sustain even when risks are explained. Simple tools such as the FIB‑4 score are helpful but not consistently used in primary care, and access to further tests like elastography varies across settings. As a result, opportunities for early intervention are often missed.

This opinion introduces ATLAS‑Liver as a conceptual reference architecture for how “agentic” artificial intelligence (AI) systems designed to coordinate multiple decision‑support tasks under clinician oversight could help strengthen existing MASLD pathways. The framework describes how components such as risk estimation, explanation of model outputs, fairness and calibration checks, curated guideline retrieval, and structured hand‑offs to clinicians might work together to support consistent and transparent triage within routine workflows. It is not a deployed or validated system, and no performance claims are made.

ATLAS‑Liver is designed to complement, not replace, established stepwise pathways like FIB‑4 followed by elastography. Its potential value lies in improving workflow integration, highlighting uncertainty, coordinating follow‑through on testing and referrals, and identifying possible equity gaps. The framework also acknowledges real‑world challenges such as governance requirements.

Substantial evidence is still needed, but ATLAS‑Liver is offered to stimulate discussion on how agentic AI might be responsibly developed and evaluated to support MASLD care in primary care environments.

## Introduction

Metabolic dysfunction-associated steatotic liver disease (MASLD) is now one of the most common chronic liver conditions worldwide, affecting up to one‑third of adults and driving substantial risks of cirrhosis, hepatocellular carcinoma, and liver‑related mortality [1,2]. Primary care remains the pivotal setting for early identification of those with MASLD-associated significant fibrosis (≥F2), a major predictor of adverse clinical outcomes [3,4] Although clinically significant portal hypertension (CSPH) is also prognostically important, it cannot be directly measured in primary care and is therefore treated in this framework only as an exploratory referral-support signal rather than a diagnostic target. Yet despite clear guideline recommendations, under‑detection persists [5]. Existing frontline tools, such as the FIB-4 index, offer simplicity but have limited discrimination, are poorly integrated into routine workflows, and provide little transparency to support clinical reasoning or shared decision‑making [6,7]. Although FIB‑4 is widely used as an initial triage tool, several additional non‑invasive tests inform MASLD fibrosis risk. The Enhanced Liver Fibrosis (ELF) test demonstrates good rule‑in performance for advanced fibrosis, vibration‑controlled transient elastography (VCTE) provides quantitative liver stiffness assessment with reasonable sensitivity and specificity, and scores such as the NAFLD Fibrosis Score (NFS) offer supplementary risk stratification. Collectively, these tools highlight both the progress and residual gaps that motivate exploration of agentic AI to support consistent, guideline‑aligned triage in primary care. These diagnostic issues may contribute to delayed diagnosis, inefficient referral pathways, and missed opportunities for early lifestyle and pharmacologic interventions. Although non-invasive tests have become the preferred first-line tools for MASLD risk stratification, liver biopsy remains the historical reference standard for diagnosing steatohepatitis and staging fibrosis [8–10]. Biopsy allows detailed histologic assessment, including inflammation, ballooning degeneration, and fibrosis stage [8]. However, its invasive nature, cost, sampling variability, and risk of complications limit its suitability for large-scale screening in primary care, reinforcing the importance of reliable non-invasive triage strategies [8,10–12].

Artificial intelligence (AI) is increasingly applied across gastroenterology, including polyp detection, image‑based classification, workflow automation, and risk prediction [13]. Recent studies have demonstrated meaningful but variable improvements in diagnostic sensitivity, efficiency, and documentation support, alongside persistent concerns regarding generalizability, interpretability, and implementation burden [14]. However, deployments to date are fragmented, unevenly distributed, and often insufficiently aligned with health system responsibilities. Many tools lack explainability, reliable calibration in real‑world populations, or governance structures that ensure oversight, safety, and equity [13,15]. Emerging agentic AI systems represent the next stage in this evolution. Agentic AI refers to software entities capable of perceiving inputs, maintaining internal states, executing goal‑directed actions, and coordinating tasks under governance constraints [16,17]. Unlike static or single‑purpose models, agentic systems are capable of orchestrating multistep tasks, dynamically retrieving evidence, monitoring uncertainty, auditing fairness, and coordinating actions while preserving clinician authority [16,17]. As such, agentic AI systems have economic, patient‑level, and clinician‑level implications. Economically, agentic AI systems have nuanced implications. While multi‑agent architectures introduce development and monitoring costs, their potential economic value lies in earlier detection of advanced fibrosis, avoidance of high‑cost late‑stage complications, reduced unnecessary referrals, and more efficient clinician time allocation. These hypotheses require formal cost‑effectiveness modeling to determine real‑world value. For clinicians, such systems may reduce cognitive load and support consistent decision‑making, while for patients, clearer explanations and coordinated follow‑through may help bridge behavioral and access barriers. In the context of ATLAS‑Liver, agency does not imply autonomy from clinicians. Rather, it denotes modular components that monitor uncertainty, retrieve relevant guidance, and propose triage actions while remaining fully subordinate to clinician authority.

We hypothesize that primary care can benefit from agentic AI systems that orchestrate multi‑step clinical tasks, provided they are embedded within robust governance, clinician‑retained authority, and careful evaluation. Orchestration, in this context, refers to the logic governing how outputs from individual agents are reconciled, including predefined priority rules (e.g., safety‑first escalation), uncertainty thresholds that trigger clinician review, and deterministic resolution of disagreement across agents. This ensures that the multi‑agent system behaves predictably and transparently rather than as a loosely coupled rules engine. Unlike many earlier decision-support tools that primarily present risk scores, agentic AI architectures are designed to retrieve evidence, monitor uncertainty, and support fairness auditing; however, the feasibility of these capabilities varies, and clinical implementation requires further research, governance, and validation. MASLD presents a particularly compelling test case for such systems. Effective triage requires synthesizing diverse electronic health record (EHR) inputs, applying guideline‑aligned thresholds, determining when to order elastography, identifying candidates for hepatology referral, and prompting timely initiation of therapy [18,19].

We present ATLAS-Liver (Adaptive Triage and Learning Agent Suite for Liver disease) as a conceptual, hypothesis-generating framework rather than an implemented, continuously learning, or validated system. In this context, “Learning” refers to a proposed future capacity for monitored updating and feedback-informed improvement under governance oversight, not autonomous self-modification in clinical use. The core of our argument is that an AI system in primary care may benefit from incorporating several key design principles, including explainability by default, embedded equity, dynamic guideline retrieval, and accountable orchestration. The rationale being that risk predictions may be more clinically useful when accompanied by patient-specific explanations that support clinician interpretation. Technologies like SHAP (Shapley Additive Explanations) can surface patient‑specific feature contributions for certain model classes (e.g., tree ensembles), which may aid clinician reasoning. However, such explanations do not guarantee causal interpretation or user trust and require careful user‑centered design and validation. Additionally, bias in AI systems can arise from limitations or imbalances in training data and therefore requires careful monitoring and governance. Agentic systems must include a dedicated fairness agent that continuously audits performance across age, sex, and ethnicity, alerting managers to drift before it becomes a clinical error. In rapidly evolving clinical environments, static software may become outdated quickly, highlighting the potential value of systems capable of incorporating updated guidance. Agentic AI must dynamically pull from current regulatory labels and clinical guidelines to ensure recommendations are always evidence-based. Finally, AI systems are best understood as tools intended to augment clinician decision-making while preserving clinician oversight and authority. As such, an orchestration layer should manage the hand-off between AI and human, ensuring that while the AI handles the heavy lifting of data synthesis, the clinician retains final authority.

ATLAS‑Liver is intended to complement, not replace, real‑world sequential pathways such as FIB‑4 followed by VCTE. These pathways mitigate some limitations of single‑step scores but remain unevenly adopted in primary care and depend heavily on clinician time, local elastography availability, and consistent follow‑through. The hypothesized value of ATLAS‑Liver lies in improving workflow integration, transparency, follow‑up coordination, and equity monitoring rather than outperforming existing tools on raw predictive accuracy. This opinion introduces a reference architecture. It does not report empirical validation, feasibility testing, usability studies, or real‑world implementation. All statements regarding potential clinical utility or system‑level impact are prospective and should be interpreted as hypotheses that require rigorous evaluation before deployment. No datasets were analyzed, and no statistical procedures were performed. All content is conceptual and based on synthesis of existing literature.

## ATLAS‑liver conceptual framework

In this manuscript, we use the term agentic to describe a set of operational characteristics that differentiate the proposed ATLAS‑Liver architecture from conventional modular clinical decision support systems. The framework is not agentic in the sense of full autonomy, but rather in its ability to operate proactively, coordinate actions across components, and adapt based on clinician interactions while remaining firmly within a human‑governed boundary. The term agentic is therefore used to denote this intermediate, carefully bounded form of autonomy, not to imply full agency or independent decision‑making.

The ATLAS‑Liver reference architecture describes how a coordinated set of risk prediction, interpretability, calibration and fairness monitoring, dynamic guideline retrieval, and orchestration agents could operate in primary care to support MASLD workflows. We delineate two domains: (a) currently feasible with existing technologies (e.g., EHR‑integrated rules engines, tree‑based or logistic models with SHAP explanations, subgroup calibration checks, and carefully curated guideline repositories) and (b) aspirational elements (e.g., continuous population‑level drift surveillance, fully dynamic retrieval‑augmented guideline updates at inference time, and automated disagreement resolution across agents). All recommendations are advisory and subordinate to clinician judgment.

**Fig 1:**
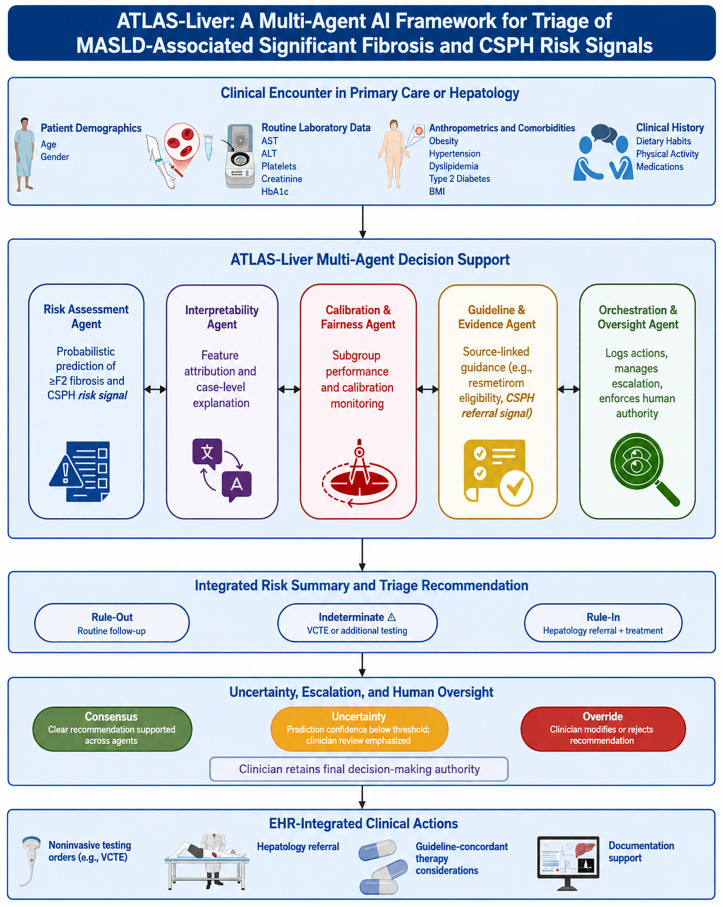
ATLAS‑Liver reference architecture (conceptual). The architecture sketches multi-agent roles (i.e., risk estimation, interpretability, calibration & fairness monitoring, guideline retrieval, orchestration) operating on routinely available clinical data to support triage for MASLD-associated significant fibrosis (≥F2). Any CSPH-related outputs are conceptual exploratory signals intended to support referral consideration rather than primary-care diagnosis or actionable determination. Outputs map to dual-threshold pathways (rule-out; indeterminate→elastography; rule-in→hepatology) and always present uncertainty. All recommendations are advisory; clinicians retain final authority. The figure depicts a conceptual design rather than a validated or implemented system.

At the center of ATLAS-Liver is the risk‑assessment agent, which uses routinely available primary care data to produce calibrated probability estimates. These outputs are paired with dual thresholds that distinguish rule‑out, indeterminate‑to‑elastography, and rule‑in‑to‑hepatology pathways, aligning directly with contemporary MASLD recommendations (**Fig 1**). The risk‑assessment agent is complemented by an explainability agent based on SHAP, which can generate concise patient‑specific rationales for each prediction.

A dedicated fairness and calibration‑monitoring agent conducts ongoing audits across key demographic and clinical subgroups. The fairness and calibration agent fits within the manager domain, enabling equity monitoring, drift detection, and quality assurance. Equity and calibration monitoring may include subgroup sensitivity, PPV, calibration‑in‑the‑large, calibration slope, and expected calibration error, with audits scheduled quarterly or semiannually. Deviations beyond predefined ranges would trigger review and potential model recalibration under governance oversight. In parallel, a guideline agent could draw from curated and version‑controlled guideline and label repositories with human oversight. Any retrieval‑augmented approach should implement source whitelisting, change logs, and a governance gate before surfaced content influences recommendations (**Fig 1**).

These agents are coordinated by an orchestration layer, which manages uncertainty, reconciles disagreements between agents, and escalates cases to clinician oversight when thresholds are crossed (**Fig 1**). All recommendations are advisory, and every action is logged to ensure traceability, safety monitoring, and governance.

The proposed orchestration follows a sequential pipeline: (1) the Risk Stratification Agent processes available EHR data and generates a probability estimate; (2) the Explainability Agent produces a patient-specific feature attribution; (3) the Guideline Retrieval Agent matches the risk profile to current clinical recommendations; and (4) the Orchestration Layer synthesizes these outputs into an alert category and, if applicable, a pre-populated clinical action. The Equity Monitoring Agent operates asynchronously on aggregated data at defined intervals (proposed: weekly) rather than at the individual encounter level. At each step, defined failure modes would trigger graceful degradation rather than unreliable outputs. For example, if required laboratory values such as AST, ALT, or platelet count are missing or outdated, the system would withhold risk classification and prompt completion of the required data rather than generate a potentially misleading estimate. If the guideline retrieval agent cannot verify that a recommendation comes from a curated and current source, the system would default to the locally approved pathway and flag the case for clinician review. If the model detects high uncertainty, calibration drift, or discordance between the risk estimate and guideline pathway, the orchestration layer would suppress automated action suggestions and escalate the case for clinician assessment. These responses are intended to preserve safety and transparency while avoiding unsupported outputs.

ATLAS‑Liver is intended as a reference architecture rather than a product, system prototype, or validated pipeline. Its purpose is to organize design principles (explainability by default, embedded equity, dynamic guidance, accountable orchestration) into a coherent blueprint that primary care settings can adapt. We explicitly separate what is feasible now and what is aspirational. Currently, it is possible to (i) conduct risk modeling using routinely available EHR data (e.g., age, AST, ALT, platelets, BMI, diabetes status, medications), (ii) align dual‑threshold triage logic with current MASLD pathways (e.g., rule‑out, indeterminate→elastography, rule‑in→hepatology), (iii) obtain local explanations for compatible models, and (iv) subgroup calibration/fairness audits [20–22]. In the future, we hope that ATLAS-Liver can facilitate (i) continuous, near‑real‑time drift detection across sites; (ii) dynamic retrieval of guidance at inference time with automated synthesis; (iii) multi‑agent disagreement resolution without human intervention. Of note, these elements require further research and governance to reach clinical readiness.

Beyond individual clinical workflow improvements, ATLAS-Liver may also have implications for population health and health equity. MASLD-associated advanced fibrosis disproportionately affects populations with limited access to hepatology subspecialty care, including rural communities and safety-net health systems. Embedding risk stratification within the primary care workflow, where many of these patients first present, could help reduce diagnostic delays linked to geographic and socioeconomic barriers. The framework’s emphasis on continuous equity monitoring also reflects a design principle aimed at detecting differential algorithm performance across demographic groups. If validated in future studies, such approaches may contribute to improving earlier identification of advanced fibrosis in underserved populations.

## Technical specificity and governance

Any system that influences triage, testing, or referral pathways would likely fall under medical device decision‑support regulations and require clear post‑deployment surveillance, defined update procedures, and transparent logs of AI‑influenced decisions. Liability must be allocated through governance structures that specify when recommendations are advisory, how overrides are documented, and how harm scenarios are reviewed. ATLAS‑Liver’s components require clear governance structures, version‑controlled clinical content, documented thresholds for calibration and fairness monitoring, and predefined escalation pathways that preserve clinician oversight. These elements represent design principles rather than validated or deployed capabilities. Importantly, governance with regard to systems like ATLAS-Liver must clarify the role of policy levers such as reimbursement reform, explainability requirements, and interoperability standards that are most impactful when tied to measurable outcomes, including elastography completion rates, reduction in inappropriate referrals, time‑to‑referral by subgroup, alert acceptance rates, and calibration drift metrics. Linking incentives to specific implementation outcomes may improve feasibility and accountability. Finally, outputs should be communicated to patients using concise, patient‑centered explanations that clarify why a recommendation was made, how uncertainty is quantified, and how the clinician supervises the system. Consent models should inform patients that AI contributes to decision support without replacing clinical judgment, and preferences regarding communication channels should be integrated into workflow.

## Implementation realism

Successful use of such an architecture would depend on careful workflow integration, mitigation of alert fatigue, clinician trust, AI literacy, interoperability, and availability of downstream resources such as elastography and hepatology services. Adoption should therefore be understood as a socio‑technical challenge requiring usability testing, governance, resourcing, and alignment of incentives; not merely technical readiness. If successfully developed and implemented, architectures such as ATLAS-Liver could influence MASLD care beyond individual prediction tasks by improving coordination of diagnostic pathways, supporting earlier identification of patients with advanced fibrosis, and promoting guideline-aligned decision support in primary care [3,4]. However, their broader impact will ultimately depend on clinician acceptance, health-system integration, and equitable access to follow-up diagnostic and specialist services.

Additional implementation challenges include data quality variability across electronic health record systems, integration with existing clinical workflows, and the need for clear governance structures to oversee model updates, monitoring, and accountability. Health systems may also face resource disparities, particularly in access to elastography or specialist hepatology services, which could influence the feasibility and equity of deployment. Addressing these challenges will require multidisciplinary collaboration among clinicians, health system leaders, informatics teams, and policymakers.

## Positioning ATLAS-liver within the emerging literature

ATLAS‑Liver builds on growing work exploring AI agents and automation in chronic liver disease, but is distinct in its primary‑care orientation, explicit governance emphasis, dual‑threshold orchestration, and integration of explainability and fairness as core architectural features. It is intended to complement, not replace, established MASLD pathways by offering a structured conceptual model for how agentic systems might support consistent, transparent triage.

## Clinical context and pathways

MASLD remains underrecognized due to asymptomatic early disease, competing clinical priorities, limited adoption of simple risk scores, incomplete access to elastography, and behavioral barriers to lifestyle modifications. These challenges reinforce the need for scalable, non-invasive triage strategies that can be embedded within primary care workflows.

A coordinated agentic architecture could support clinicians by surfacing guideline-aligned actions, clarifying rationale, facilitating follow-through, and reinforcing established sequential pathways such as FIB-4 followed by elastography where appropriate. Such systems may help address common attrition points in the MASLD care cascade, including under-recognition of at-risk individuals, delays in non-invasive testing, and incomplete referral to hepatology services.

If successfully developed and implemented, systems such as ATLAS-Liver could contribute to shifting fibrosis detection from a predominantly reactive process in specialty settings toward earlier risk stratification within routine primary care workflows. Earlier identification of patients with clinically significant fibrosis may enable more timely lifestyle and pharmacologic interventions as therapeutic options continue to expand. At the population level, improved identification of fibrosis risk within primary care could also support more consistent application of guideline-based care. However, these potential benefits remain hypothetical and will require rigorous empirical evaluation in real-world settings.

## Challenges

Real-world deployment of a system such as ATLAS-Liver would face several structural, organizational, and regulatory challenges beyond the performance of its predictive components. Interoperability remains a key constraint, as integration with heterogeneous electronic health record environments depends on variable levels of FHIR and API maturity across health systems. Successful implementation also depends heavily on institutional readiness, including workflow redesign, clinician engagement, and the presence of clinical champions to support adoption of decision-support tools. Clinician training represents an additional requirement, as familiarity with AI-assisted workflows and interpretation of explainability outputs varies widely in primary care. Regulatory considerations may also influence deployment, particularly where components could fall under Software as a Medical Device oversight depending on functionality and autonomy. Sustaining governance structures for monitoring safety, calibration, and equity introduces further operational demands. Finally, local calibration of thresholds and referral pathways would be necessary to account for differences in disease prevalence, specialist access, and health system capacity. Together, these factors highlight that implementation represents a socio-technical challenge requiring coordinated attention to infrastructure, workflow integration, governance, and patient trust.

## Limitations and evaluation roadmap

This opinion presents a conceptual architecture without empirical validation, feasibility studies, or real‑world performance data, and no claims of effectiveness or readiness are made. A rigorous evaluation pathway would be required before considering clinical use. In addition, CSPH is referenced only as an aspirational exploratory signal. In primary care, CSPH cannot be directly measured, and any estimate would rely on probabilistic modeling of routinely collected parameters. For this reason, CSPH-related outputs should be viewed as exploratory signals for referral consideration rather than actionable diagnostic determinations. Finally, the impact of ATLAS-Liver must be tempered because many patients identified with MASLD do not sustain recommended lifestyle modifications despite repeated counseling. Improved detection alone is insufficient, but agentic AI could potentially assist by generating patient‑friendly explanations, supporting follow‑through reminders, coordinating diet and lifestyle referrals, and identifying individuals at risk of non‑adherence for proactive outreach.

## Call to action

Real‑world progress will require socio‑technical work across policy, human factors, and engineering. We recommend that health systems and regulators approve only decision‑support tools with documented data provenance, model cards, uncertainty reporting, and equity monitoring plans; support standards‑based integration so primary care can operationalize guideline‑aligned MASLD pathways consistently, require staged evidence (retrospective simulation → usability → pilot) and post‑deployment monitoring for calibration drift, alert burden, and equity impacts; provide protected time and micro‑learning on AI use, limitations, and overrides; design incentives that reward appropriate follow‑through (e.g., elastography completion after indeterminate stratification) rather than raw referral volume; and ensure elastography and hepatology resources can absorb triage‑driven referrals to avoid access bottlenecks and inequities.

## Conclusion

MASLD-associated fibrosis may be more effectively managed when identified earlier in the disease course. Agentic AI offers a promising but unproven path to strengthen MASLD pathways in primary care. ATLAS‑Liver is offered as a reference architecture to guide hypothesis‑generating evaluation and responsible design. Demonstrating clinical usefulness, safety, and equitable impact will require staged, transparent evidence generation and strong governance.

## References

[1] Feng G, Targher G, Byrne CD, Yilmaz Y, Wai-Sun Wong V, Adithya Lesmana CR, et al. Global burden of metabolic dysfunction-associated steatotic liver disease, 2010 to 2021. JHEP Rep. 2024;7(3):101271. doi: 10.1016/j.jhepr.2024.101271 39980749 PMC11840544

[2] Kan C, Zhang K, Wang Y, Zhang X, Liu C, Ma Y, et al. Global burden and future trends of metabolic dysfunction-associated Steatotic liver disease: 1990-2021 to 2045. Ann Hepatol. 2025;30(2):101898. doi: 10.1016/j.aohep.2025.101898 40057034

[3] Lionis C, Papadakis S, Anastasaki M, Aligizakis E, Anastasiou F, Francque S, et al. Practice recommendations for the management of MASLD in primary care: consensus results. Diseases. 2024;12(8):180. doi: 10.3390/diseases12080180 39195179 PMC11353634

[4] Allen AM, Charlton M, Cusi K, Harrison SA, Kowdley KV, Noureddin M, et al. Guideline-based management of metabolic dysfunction-associated steatotic liver disease in the primary care setting. Postgrad Med. 2024;136(3):229–45. doi: 10.1080/00325481.2024.2325332 38465573

[5] Huang DQ, Wong VWS, Rinella ME, Boursier J, Lazarus JV, Yki-Järvinen H, et al. Metabolic dysfunction-associated steatotic liver disease in adults. Nat Rev Dis Primers. 2025;11(1):14. doi: 10.1038/s41572-025-00599-1 40050362

[6] European Association for the Study of the Liver (EASL), European Association for the Study of Diabetes (EASD), European Association for the Study of Obesity (EASO). EASL-EASD-EASO clinical practice guidelines on the management of metabolic dysfunction-associated steatotic liver disease (MASLD). Obes Facts. 2024;17(4):374–444. doi: 10.1159/000539371 38852583 PMC11299976

[7] Gupta U, Ruli T, Buttar D, Shoreibah M, Gray M. Metabolic dysfunction associated steatotic liver disease: current practice, screening guidelines and management in the primary care setting. Am J Med Sci. 2024;367(2):77–88. doi: 10.1016/j.amjms.2023.11.007 37967750

[8] Rinella ME, Neuschwander-Tetri BA, Siddiqui MS, Abdelmalek MF, Caldwell S, Barb D, et al. AASLD Practice Guidance on the clinical assessment and management of nonalcoholic fatty liver disease. Hepatology. 2023;77(5):1797–835. doi: 10.1097/HEP.0000000000000323 36727674 PMC10735173

[9] EASL-EASD-EASO. EASL-EASD-EASO clinical practice guidelines on the management of metabolic dysfunction-associated steatotic liver disease (MASLD): Executive summary. Diabetologia. 2024;67(11):2375–92. 38869512 10.1007/s00125-024-06196-3 PMC11519095

[10] Castera L, Friedrich-Rust M, Loomba R. Noninvasive assessment of liver disease in patients with nonalcoholic fatty liver disease. Gastroenterology, 2019;156(5):1264–81.e4. 30660725 10.1053/j.gastro.2018.12.036 PMC7505052

[11] Ratziu V, Charlotte F, Heurtier A, Gombert S, Giral P, Bruckert E, et al. Sampling variability of liver biopsy in nonalcoholic fatty liver disease. Gastroenterology. 2005;128(7):1898–906. doi: 10.1053/j.gastro.2005.03.084 15940625

[12] Chalasani N, Younossi Z, Lavine JE, Diehl AM, Brunt EM, Cusi K, et al. The diagnosis and management of non-alcoholic fatty liver disease: practice Guideline by the American Association for the Study of Liver Diseases, American College of Gastroenterology, and the American Gastroenterological Association. Hepatology. 2012;55(6):2005–23. doi: 10.1002/hep.25762 22488764

[13] Topol EJ. As artificial intelligence goes multimodal, medical applications multiply. Science. 2023:eadk6139. 10.1126/science.adk6139 37708283

[14] Sarkar U, Bates DW. Using artificial intelligence to improve primary care for patients and clinicians. JAMA Intern Med. 2024;184(4):343–4. doi: 10.1001/jamainternmed.2023.7965 38345801

[15] Samek W, Müller K-R. Towards explainable artificial intelligence. Explainable AI: interpreting, explaining and visualizing deep learning. Springer; 2019. p. 5–22.

[16] Huang K. AI agents in healthcare. Agentic AI: theories and practices. Springer; 2025. p. 303–21.

[17] Karunanayake N. Next-generation agentic AI for transforming healthcare. Informatics and Health. 2025;2(2):73–83. doi: 10.1016/j.infoh.2025.03.001

[18] Decharatanachart P, Chaiteerakij R, Tiyarattanachai T, Treeprasertsuk S. Application of artificial intelligence in non-alcoholic fatty liver disease and liver fibrosis: a systematic review and meta-analysis. Ther Adv Gastroenterol. 2021;14:17562848211062807. doi: 10.1177/17562848211062807 34987607 PMC8721422

[19] Singh Y, Hathaway QA, Vera-Garcia DV, Povero D, Salehi S, Wei Y, et al. Artificial Intelligence-based agents in chronic liver disease: transforming diagnostic and therapeutic workflows through clinical decision-making. npj Gut Liver. 2025;2(1). doi: 10.1038/s44355-025-00049-5

[20] Njei B, Osta E, Njei N, Al-Ajlouni YA, Lim JK. An explainable machine learning model for prediction of high-risk nonalcoholic steatohepatitis. Sci Rep. 2024;14(1):8589. doi: 10.1038/s41598-024-59183-4 38615137 PMC11016071

[21] Njei B, et al. Fibrox: an explainable AI model for accurate prediction of advanced liver fibrosis and cardiovascular mortality in MASLD. Gastroenterology. 2025;169(1):S-131-S-132.

[22] Njei B, Al-Ajlouni YA, Lemos SY, Ugwendum D, Njei N, Al Ta’ani O, et al. AI-based models for risk prediction in MASLD: a systematic review. Dig Dis Sci. 2026;71(5):1987–2014. doi: 10.1007/s10620-025-09499-6 41231418

